# Duration of the War from the Standpoint of Vital Statistics

**Published:** 1916-01

**Authors:** 


**A Monthly Review of Medicine and Surgery**

EDITOR AND PUBLISHER, DR. A. L. BENEDICT, 228 Summer St., corner of Elmwood Ave., Buffalo, (Address for all communications. Please make personal and telephone calls before 1 P. M.)

Third, in age, on the western continent. Independent, but supports professional organization. News limited mainly to Western N. Y. and Penn.

Subscription $2.00 a year; $3.00 for two years in advance. Changes and errors in address or failure or duplication of delivery, should be reported immediately.

Yearly Volume 71. JANUARY, 1916. No. 6

### Duration of the War From the Standpoint of Vital Statistics.

Sufficient time has elapsed to indicate with considerable probability that the present European War is, like the Boer War, though for different reasons, to be one of "attrition." Whatever strategic expectation there might have been at the beginning of a brief campaign overwhelming relatively unprepared opponents, has been dismissed. In all past wars, the forces were comparatively small, and armies manouvered in ample space as detached bodies. Psychologically, a whole army was inspired by success or dispirited by reverses. At the beginning of this war, the question was raised whether, under these circumstances, an army could be routed or demoralized as it had in the past. This question seems to have been answered in the negative. Local victories may be won, and superior driving force may affect a whole line. Unquestionably, too, the war will end with the recognition of defeat before an army has actually been reduced to insignificant numbers. But there will be no actual rout, such as occurred at Waterloo, and probably no such lack of material and food as determined the outcome at Yorktown or at Appomattox. Neither does it seem probable that, so far as the main lines of battle are concerned, there will be any such retreats with avoidance of actual hostilities as have marked most previous wars.

The following statistics were compiled from the U. S. census of 1900, for native whites born of native whites, in the registration area, the total population of the series being a little over 3 million. These statistics differ somewhat from those of the actual population of the registration area, on account of the distortion due to immigration, which increases the proportion of male adults. They probably apply fairly well to conditions existing in the European countries, though they do not exactly represent conditions present on account of differences of birth rate, infantile and general mortality, and emigration.

| | |
|---|---|
| Under 1 year | 2.4% |
| 1-4 years | 8.8 |
| 15-24 years | 18.2 |
| 5-14 years | 20.6 |
| | 50. % |
| 25-34 years | 15.1 |
| 35-44 years | 11.9 |
| 45-64 years | 16.4 |
| Over 65 years | 6.2 |
| Unknown | .4 |

In any large population, the sexes are nearly equally divided, there being a slight excess of males in territories toward which immigration flows, and a slight excess of females in countries subject to emigration. Indeed, in recently settled territories, the excess of males and of young adults is very marked.

The military strength of a community varies widely according to circumstances. In a small savage or even relatively civilized community, a brief defense against an invading force may conceivably bring into action all but the very young and the very old and the sick and the crippled, without regard to sex. 75 or 80% of the community may thus be reckoned as military forces, and this has been the actual experience in the colonial period of our country, while a close approach to it is noted in several localities during the Revolution. 18% of an average community consists of males over 20 and under 45. Just about 25% represents the entire male portion of the community over 20, with a reasonable allowance for incapacity at ages beyond 45. From these last two numbers must be subtracted those incapacitated at earlier ages, but this substraction may be balanced by placing the age of military service at 19 or possibly at 18.

Under modern conditions, practically no allowance can be made for female service, such women as are available, even with no regard for sentiment, being required to take the place of men in various occupations and for nursing and clerical duties and the like in connection with the army. Thus, from 17 to 25% of the population is the largest estimate of military force possible. With the present requirements for food, transportation, manufacture of arms, ammunition and other military supplies, it is scarcely conceivable that these percentages are actually available for direct military purposes, even with the sacrifice of industry, education and everything else, and the utmost utilization of women in positions normally held by men. In the American Revolution, almost exactly 11% of the total population served, beside a considerable number in temporary and informal defense, but the terms of service were for the most part brief, for any given man or unit. In the War of 1812, about 8% of the population served, and in the Civil War, about 11% for the entire, though divided country.

Other experience has shown that a country may maintain its industries and normal life to a fairly satisfactory degree, while putting 10% of its population in the field at one time or another. Considering, on the one hand, the more systematic arrangements for thus conducting the economic life of a nation during a protracted war and, on the other, the greater number of men absolutely required for the maintenance of supplies for military use, it is a reasonable estimate that 15% of the total population may be put into service. About 1-8 of the Union troops died, and more than a sixth of the Confederate. Obviously, however, in a determined contest, without failure of supplies such as really caused the end of the Civil War, the forces might be reduced to the lowest considerable fighting unit before reaching a decision. It must also be considered that nearly 1% of a population will, yearly, be available as the children become mature, and that this number will continue to represent 1% of the original population for say 18 years, before the effect of war on reproduction will become manifest.

It will simplify the problem to consider only the central, Teutonic forces, since their opponents have a considerably larger reserve from which to draw, and since the Turkish and Balkan forces will undoubtedly be engaged in contests on a different field. Germany has a population of about 65 million, Austria-Hungary of about 50 million, total 115 million. Available military strength on 15% estimate, 17 million plus, with annual increment of 1 million. The total losses for the first year, estimated proportionately to reports for the first nine months, in killed, wounded and prisoners, allowing for the return of about half the wounded to the ranks, were about 8½ million. The net loss would be reduced to 7½ million by the increment of boys. At this rate, granted that neither side falls short of ammunition, and that no considerable victory is won by either side, the war must cease by the effects of attrition within three years. It is altogether likely, however, that mutual conservation of men will greatly reduce the losses of all kinds on both sides. Thus as a war of attrition alone, the present conflict may be protracted for at least five years, and it is, of course, conceivable that the war will not continue to be one of attrition but that new destructive devices or, on the other hand, psychologic tendencies toward defeat or triumph, may develop on either side, so as to bring about a speedier termination.

